# Elevated plasma level of PAI-1 is associated with severe COVID-19

**DOI:** 10.1038/s41598-025-06517-5

**Published:** 2025-08-04

**Authors:** Susumu Fukahori, Yurika Kawazoe, Jung Yeon Han, Narasaiah Kolliputi, Kaitlyn Lezama, Rajesh Kumar, Hiroshi Mukae, Richard F. Lockey, Iset Medina Vera, Kami Kim, Seong H. Cho

**Affiliations:** 1 Division of Allergy and Immunology, Department of Internal Medicine, USF Morsani College of Medicine, Tampa, FL USA; 2 Department of Respiratory Medicine, Nagasaki University Hospital, Nagasaki, Japan; 3 Clinical Research Center, Nagasaki University Hospital, Nagasaki, Japan; 4 Division of Allergy and Immunology, Department of Pediatrics, Northwestern University and the Ann and Robert H. Lurie Children’s Hospital, Chicago, IL USA; 5 Department of Respiratory Medicine, Nagasaki University Graduate School of Biomedical Sciences, Nagasaki, Japan; 6 Division of Infectious Disease, Department of Internal Medicine, USF Morsani College of Medicine, Tampa, FL USA; 7 Tampa General Hospital, Tampa, FL USA

**Keywords:** COVID-19, PAI-1, Biomarker, Prognostic factor, Prognostic markers, Coagulation system, Viral infection, Translational research

## Abstract

Evidence indicates endothelial dysfunction in severe coronavirus disease (COVID-19). Plasminogen activator inhibitor-1 (PAI-1) is a marker of endothelial injury and could be a prognostic marker for COVID-19-related hospitalization and outcomes. The association between PAI-1 levels and the severity of COVID-19-related outcomes was investigated in this study. This single-center retrospective chart review included 113 hospitalized adults from 6.29.2020 to 8.1.2021 with confirmed COVID-19. Plasma PAI-1 levels were measured by ELISA. The primary endpoint was the difference in PAI-1 levels between severe and non-severe COVID-19 groups. Severe COVID-19 was defined as the need for ventilator assistance and/or death. The mean age was 60.78 (22 to 103, SD ± 16.93), and 52 were female and 63 male. There was a significant positive correlation between age and PAI-1 levels. PAI-1 levels were significantly higher in patients with hyperlipidemia. PAI-1 levels in patients requiring ventilator assistance and who died versus those who did not are significantly higher. High plasma PAI-1 levels are associated with severe COVID-19, defined as requiring ventilator use and/or death. Thus, PAI-1 may be a biological marker for severe COVID-19.

## Introduction

The COVID-19 pandemic, caused by the SARS-CoV-2 virus, has posed both medical and social challenges. Although many infected individuals experience only mild symptoms, those with high-risk factors such as older age or pre-existing medical conditions (e.g., diabetes, kidney disease) may develop severe pneumonia or acute respiratory distress syndrome (ARDS)^1^. Hence, biomarkers that can accurately and simply predict severe disease would facilitate the efficient allocation of scarce medical resources, enable timely and appropriate treatment during the initial stages of disease progression, and alleviate the strain on the healthcare system.

Thrombosis is a common complication of patients with severe COVID-19 ^2^^3^, . Prior research indicates that the disparity in fibrinolytic activators and inhibitors among patients with severe COVID-19 may be related to elevated levels of PAI-1. This may contribute to the severity of the disease^4,5^. Likewise, higher levels of PAI-1 are associated with adverse clinical outcomes, as reported by Hirai^6^. It is noteworthy that PAI-1 levels tend to increase under various conditions, including aging, cardiovascular disease, COPD, smoking, and obesity^7–11^. These conditions are also known to be poor prognostic indicators for COVID-19^12^.

Therefore, the current investigation sought to ascertain whether plasma levels of PAI-1 are independently linked to the severity of COVID-19, after adjusting for age, BMI, comorbidities, and smoking status.

## Methods

### Study design and data source

This is a single-center retrospective chart review of patients with COVID-19 admitted to the Tampa General Hospital (TGH), Tampa, FL, USA, between June 29, 2020, and August 1, 2021, in whom SARS-CoV-2 was detected by PCR. There were no exclusion criteria. Plasma samples from 113 patients hospitalized with COVID-19 were utilized. Blood samples were collected using ethylenediaminetetraacetic acid-treated tubes in the early morning on the following day of admission. After completion of hematology testing ordered by the clinician, the remaining plasma was stored at − 80 °C until testing. PAI-1 plasma level at the initial visit were measured using ELISA (Assay Pro) as previously described^13^. All records, including epidemiological, clinical, laboratory, and outcome data, were obtained from the electronic medical records. This study complied with all relevant ethical regulations and was approved by the University of South Florida, Tampa, FL, IRB (HUM00179409). For COVID-19 samples, the University of South Florida Institutional Review Board (IRB) waived the requirement for informed consent given the discarded nature of the samples. The primary endpoint was the comparison of PAI-1 levels between the severe and non-severe COVID-19 groups. Severe illness due to COVID-19 was defined as the need for ventilator assistance and/or death.

### Statistical analysis

Categorical variables are summarized as counts (percentages). Continuous variables are presented as mean ± standard deviation (SD) for normally distributed samples and as median and interquartile range (IQR) for those without normal distribution. The differences in mortality rates between males and females, as well as the impact of each comorbidity on mortality rates, are assessed using a chi-square test. The long right-side distribution of PAI-1 values necessitated log transformation. As log-transformed PAI-1 displays a normal distribution pattern, the variable logPAI-1 was subsequently employed in further analyses.

Differences in PAI-1level between the groups divided by sex, smoking status, presence or absence of each comorbidity, severity defined as requiring ventilator management, and survival status were tested using the Mann–Whitney U test. The correlation between logPAI-1 and age and the correlation between logPAI-1 and BMI were compared using Pearson’s correlation coefficient. The relationship between sex, smoking history, and comorbidity with ventilator management and death was assessed by calculating odds ratios (ORs) and 95% confidence intervals (CI) using univariate logistic regression analysis. To assess the relationship between logPAI-1, ventilator requirement, and mortality, five models with varying adjustment variables and employed multivariate logistic regression analysis to calculate odds ratios and their corresponding 95% confidence intervals were developed. Adjusted variables included in the models were age, BMI, smoking status, and comorbidities. As a supplementary analysis, a stratified logistic regression analysis was performed to evaluate the association between logPAI-1 levels, ventilator management requirements, and death for each comorbidity. Statistical analyses were conducted using JMP Pro, Version 17.2 (SAS). The interpretation of P-values should serve as a reference, as the study is exploratory.

### Ethics and dissemination

The study protocol was approved by the Clinical Research Review Board of the University of South Florida (Protocol HUM00179409) and conducted in accordance with the principles of the Declaration of Helsinki.

## Results

### Demographic and clinical characteristics of the study participants

Individuals admitted with COVID-19 to TGH were managed by a multidisciplinary team comprised of hospitalists, pulmonary/critical care specialists, infectious disease specialists, nursing, respiratory therapy, pharmacy, and case management. Thus, all patients with severe COVID-19 were managed with internally consistent approaches that evolved as additional guidelines and treatments became available. The period of this study overlapped with the original, alpha, and delta waves of infection in the Tampa Bay region.

Table 1 presents participants’ basic demographics. The 113 subjects evaluated in this study had an average age of 60.6 years, with a standard deviation of 17.0. Of these, 53.9% (61) were male, 35.4% (40) Hispanic, 34.5% (39) Caucasian, and 23.9% (27) Black. 7.1% were current smokers, 25.7% former smokers, and 67.3% non-smokers (8, 29, and 76 patients, respectively). The mean BMI was 29.5 with a standard deviation of 8.1. 21.2% required ventilator management (24) and the overall mortality rate was 19.5% (22).

Among the individuals included in the study, 34.5% (39) had type I or II diabetes mellitus, 19.4% (22) coronary artery disease, 21.2% (24 ) hyperlipidemia, 52.2% (59) hypertension, 14.1% (16 ) chronic kidney disease, 10.6% (12 ) had undergone organ transplantation of any kind, 6.1% (7) Chronic Obstructive Pulmonary Disease (COPD), 2.7% (3) cirrhosis, 10.6% (12 ) cancer, 2.7% (3 ) dementia, and 35.4% (40) obesity, as defined by a body mass index greater than 30.

### Correlation coefficient between LogPAI-1 and age, LogPAI-1 and BMI

**Fig. 1:**
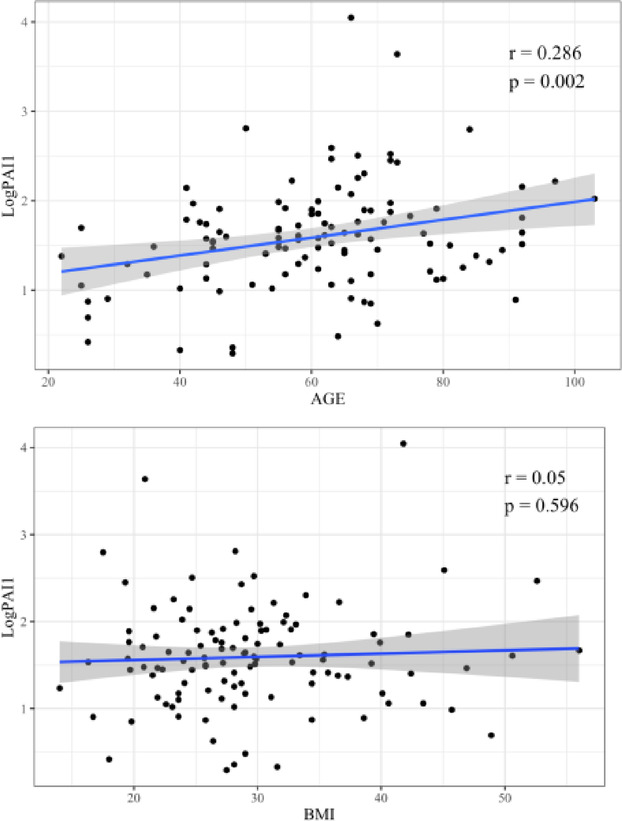
Correlation between PAI-1 levels and age/BMI. (**a**) shows the scatter plot and linear regression analysis of logPAI-1 with age. (**b**) shows the scatter plot and linear regression analysis of logPAI-1 with BMI.

Figure 1a shows the correlation between logPAI-1 levels and age, and Fig. 1b the correlation between logPAI-1 levels and BMI, as determined using simple linear regression analysis. Age was positively correlated with logPAI-1 (*r* = 0.286, *p* < 0.002).

In contrast, no significant correlation was found between logPAI-1 and BMI.

### PAI-1 levels across different categories

Table 2 shows the median and interquartile range (IQR) of the PAI-1 levels in various categories, including sex, smoking status, and comorbidities. Notably, PAI-1 levels were higher in patients who required ventilator assistance, those who died, and those with hyperlipidemia.

**Table 1:** Participants’ basic demographics.

Characteristic	
Age yr mean ± SD	60.6 ± 17.0
Age group-no. (%)	
< 40	10 (8.9)
40–50	19 (16.8)
50–60	20 (17.7)
60–70	35 (31.0)
70–80	15 (13.3)
80<	14 (12.4)
Male sex-no. (%)	61 (53.9)
Race or ethnicity group - no. (%)	
White	39 (34.5)
Black	27 (23.9)
Hispanic	40 (35.4)
Others	7 (6.2)
Smoking status - no. (%)	
Non-smoker	76 (67.3)
Former-smoker	29 (25.7)
Current-smoker	8 (7.1)
Body-mass index	29.5 ± 8.1
Number of patients requiring ventilator management- no. (%)	22 (19.5)
Number of patients who have died- no. (%)	24 (21.2)
Cormorbidities -no. (%)	
Diabetes	39 (34.5)
Coronary artery disease	22 (19.4)
Hyperlipidemia	24 (21.2)
Hypertension	59 (52.2)
Chronic kidney disease	16 (14.1)
Post transplantation	12 (10.6)
COPD	7 (6.1)
Asthma	13 (11.5)
Liver cirrosis	3 (2.7)
Cancer	12 (10.6)
Dementia	3 (2.7)
Obesity	40 (35.4)

**Table 2:** PAI-1 levels in various categories.

	PAI-1 (IQR)	*p* value
Gender		
Male	4.76 (3.47 to 6.67)	
Female	4.91 (3.34 to 6.59)	0.73
Smoking status		
Never	4.59 (3.34 to 5.94)	
former	6.22 (3.72 to 7.45)	
current	4.16 (2.73 to 5.08)	
Ventilated (+)	5.45 (4.47 to 9.15)	
Ventilated (-)	4.57 (3.24 to 6.57)	< 0.05
Died (+)	5.77 (4.68 to 8.86)	
Died (-)	4.57 (3.24 to 6.40)	< 0.05
Comorbidities		
COPD (+)	5.13 (2.77 to 12.25)	
COPD (-)	4.79 (3.58 to 6.54)	0.76
Diabetes (+)	4.83 (3.09 to 6.36)	
Diabetes (-)	4.78 (3.64 to 6.72)	0.57
Coronary artery disease (+)	4.68 (3.32 to 6.70)	
Coronary artery disease (-)	4.87 (3.63 to 6.61)	0.7
Hyperlipidemia (+)	5.71 (4.24 to 8.46)	
Hyperlipidemia (-)	4.57 (3.17 to 6.29)	< 0.05
Hypertension (+)	5.13 (4.07 to 6.80)	
Hypertension (-)	4.50 (3.10 to 6.14)	0.1
Chronic kidney disease (+)	3.81 (2.80 to 5.76)	
Chronic kidney disease (-)	4.87 (3.69 to 6.71)	0.07
Post transplantation (+)	3.87 (3.27 to 5.52)	
Post transplantation (-)	4.87 (3.65 to 6.67)	0.16
Obesity (+)	5.01 (3.70 to 7.06)	
Obesity (-)	4.70 (3.40 to 6.17)	0.52

The values of the PAI-1 were represented as median (25-75% IQR).

Differences between groups were tested by hypothesis testing using the Mann-Whitney U test.

**Table 3:** The rates and odds ratios for mechanical ventilation (a). The rates and odds ratios for death (b).

**(a)**					
		Number of patients requiring ventilator management (%)	Odds Ratio	95% CI	*p* value
Gender					
male		17 (27.9)	2.48	0.94 to 6.57	0.06
female		7 (13.5)			
Smoking Status					
Never		17 (22.4)			
Former		6 (20.7)			
Current		1 (12.5)			
Comorbidity					
Hypertension	(+)	14 (23.7)	1.37	0.55 to 3.41	0.5
	(-)	10 (18.52)			
Coronary artery disease	(+)	5 (22.7)	1.11	0.36 to 3.41	0.85
	(-)	19 (20.9)			
Hyperlipidemia	(+)	5 (20.8)	0.98	0.41 to 2.34	0.96
	(-)	19 (21.4)			
Diabetes	(+)	11 (28.2)	1.84	0.74 to 4.62	0.2
	(-)	13 (17.6)			
COPD	(+)	3 (42.9)	3.04	0.63 to 14.6	0.18
	(-)	21 (19.8)			
Asthma	(+)	3 (23.1)	1.12	0.28 to 4.47	0.86
	(-)	21 (21.0)			
Liver Cirrhosis	(+)	1 (33.3)	1.89	0.16 to 21.79	0.62
	(-)	23 (20.9)			
Any form of malignancy	(+)	0 (0)	N/C	N/C	N/C
	(-)	24 (23.8)			
Dementia	(+)	0 (0)	N/C	N/C	N/C
	(-)	24 (21.8)			
Obesity	(+)	10 (25.0)	1.4	0.56 to 3.53	0.47
	(-)	14 (19.8)			

**(b)**					
		Number of death (Mortality rate %)	Odds Ratio	95% CI	*p* value
Gender					
Male		15 (24.6)	2.09	0.78 to 5.62	0.13
Female		7 (13.5)			
Smoking Status					
Never		14 (18.4)			
Former		6 (20.7)			
Current		2 (25.0)			
Comorbidity					
Hypertension	(+)	9 (15.3)	0.56	0.22 to 1.46	0.34
	(-)	13 (24.1)			
Coronary artery disease	(+)	8 (36.4)	3.14	1.11 to 8.88	< 0.05
	(-)	14 (15.4)			
Hyperlipidemia	(+)	5 (20.8)	1.11	0.36 to 3.41	1
	(-)	17 (19.1)			
Diabetes	(+)	9 (23.1)	1.41	0.54 to 3.66	0.62
	(-)	13 (17.6)			
COPD	(+)	4 (57.1)	6.52	1.34 to 31.66	< 0.05
	(-)	18 (17.0))			
Asthma	(+)	2(15.4)	0.72	0.15 to 3.55	1
	(-)	20 (20.0)			
Liver Cirrhosis	(+)	2 (66.7)	9	0.78 to 104.18	0.1
	(-)	20 (18.2)			
Any form of malignancy	(+)	2 (16.7)	0.81	0.16 to 3.99	1
	(-)	20 (19.8)			
Dementia	(+)	2 (66.7)	9	0.78 to 104.18	0.1
	(-)	20 (18.2)			
Obesity	(+)	17 (23.3)	0.47	0.16 to 1.39	0.22
	(-)	5(12.5)			

### Analysis of mortality rates and the need for mechanical ventilation by sex, smoking status, and comorbidities

Table 3 displays the rates and odds ratios for death or the need for mechanical ventilation among patients classified according to sex, smoking status, and presence or absence of specific comorbidities.

The mortality rate of individuals with coronary artery disease was 36.4%, and the odds ratio (OR) for death was 3.14 (95% CI, 1.11 to 8.88). Furthermore, the mortality rate of patients with COPD was 57.1%, with an OR for death of 6.52 (95% CI, 1.34 to 31.66). Regarding the other previously described risk factors, there were no differences in mortality rates and ORs for death in this sample. Additionally, there were no differences in the proportion of patients and the OR for requiring mechanical ventilation in all categories and comorbidities (data not shown).

### Levels of PAI-1 associated with worse outcomes after adjusted for age, sex, BMI, and comorbidities

Multivariate logistic regression analysis was conducted to examine the relationship between logPAI-1 levels and patient outcomes and the results are presented in Table 4.

**Table 4:** The association between PAI-1 levels and mechanical ventilation/death.

Ventilation				
		OR	95% CI	p value
LogPAI-1 (+ 1)	model 1	3.02	1.28 to 7.98	< 0.05
	model 2	3.12	1.28 to 8.43	< 0.05
	model 3	3.12	1.28 to 8.45	< 0.05
	model 4	3.35	1.37 to 9.13	< 0.05
	model 5	3.42	1.40 to 9.32	< 0.05

Survival				
		OR	95% CI	p value
LogPAI-1 (+ 1)	model 1	2.78	1.18 to 7.32	< 0.05
	model 2	2.89	1.19 to 7.93	< 0.05
	model 3	2.94	1.20 to 8.29	< 0.05
	model 4	3.13	1.27 to 8.91	< 0.05
	model 5	3.16	1.28 to 9.05	< 0.05

Model 1: adjusted for age, sex, and BMI.

Model 2 was adjusted for variables in Model 1 plus respiratory disease.

Model 3 was adjusted for variables in Model 2 plus cardiovascular disease.

Model 4 was adjusted for variables in Model 3 plus diabetes.

Model 5 was adjusted for variables in Model 4 plus hyperlipidemia.

Model 1 was developed to consider age, gender, and body mass index (BMI). Model 1 demonstrated a relationship between logPAI-1 levels and the requirement for mechanical ventilation support (OR 3.02, 95% CI 1.28 to 7.98) and mortality (OR 2.78, 95% CI 1.18 to 7.32). Model 2 was adjusted for the variables in Model 1 as well as for respiratory diseases. Model 2 showed that logPAI-1 levels were associated with the need for mechanical ventilation support (OR 3.12, 95% CI 1.28 to 8.43) and death (OR 2.89, 95% CI 1.19 to 7.93). Model 3 was adjusted for the variables in Model 2 as well as for cardiovascular disease. Model 3 revealed that logPAI-1 levels were associated with the need for mechanical ventilation support (OR 3.12, 95% CI 1.28 to 8.45) and death (OR 2.94, 95% CI 1.20 to 8.29). Model 4 was adjusted for the variables in Model 3 and for diabetes. Model 4 showed that logPAI-1 levels were associated with the need for mechanical ventilation support (OR 3.35, 95% CI 1.37 to 9.13) and death (OR 3.13, 95% CI 1.27 to 8.91). Model 5 was adjusted for the variables in Model 4 and hyperlipidemia. Model 5 indicated that logPAI-1 levels were associated with the need for mechanical ventilation (OR 3.42, 95% CI 1.40 to 9.32) and death (OR 3.16, 95% CI 1.28 to 9.05). After accounting for age, sex, BMI, and comorbidities, logPAI-1 levels remained independently associated with critical illness.

### Predictive value of logPAI-1 levels for outcomes for patients with comorbidities

**Fig. 2:**
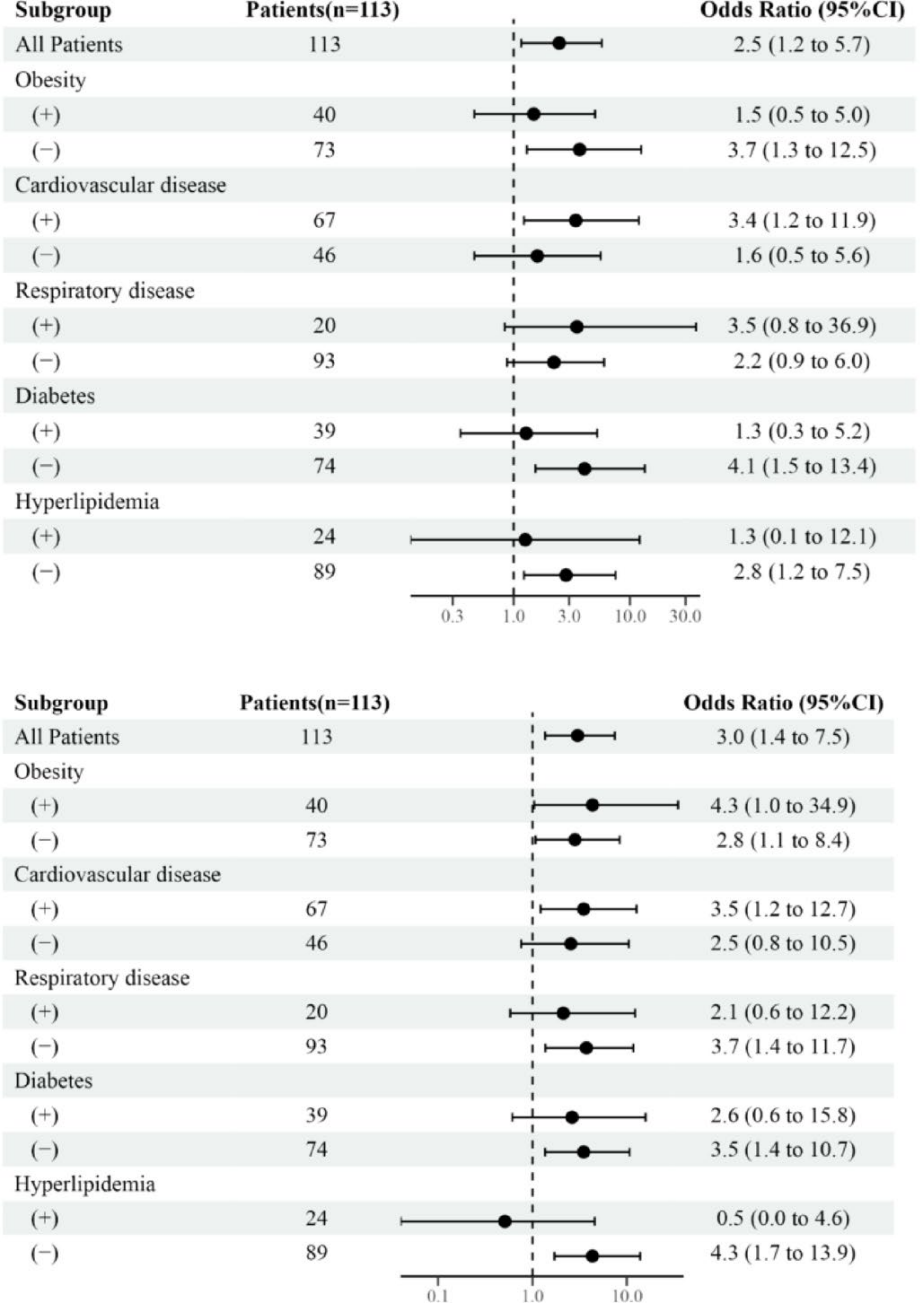
A forest plot depicting the relationship between logPAI-1 levels and outcomes in patients with and without comorbidities. (**a**) shows Odd Ratio (ORs) for requiring mechanical ventilation support in patients with and without comorbidities. (**b**) shows ORs for death in patients with and without comorbidities.

Univariate logistic regression analysis of the associations between logPAI-1 levels and outcomes in patients with and without comorbidities is shown in Fig. 2. Univariate analysis revealed that 1 unit of logPAI-1 increase yielded ORs for requiring mechanical ventilation support of 3.7 (95% CI, 1.3 to 12.5) without obesity, 3.4 (95% CI, 1.2 to 11.9) with cardiovascular disease (including both hypertension and coronary artery disease), 4.1 (95% CI, 1.5 to 13.4) without diabetes, and 2.8 (95% CI, 1.2 to 7.5) without hyperlipidemia. There was a significant correlation between logPAI-1 levels and mechanical ventilation support requirements in patients with cardiovascular disease, and without obesity, diabetes, and hyperlipidemia.

Also, 1 unit of logPAI-1 increase yielded ORs for death of 4.3 (95% CI, 1.04 to 34.9) in patients with obesity, 2.8 (95% CI, 1.1 to 8.4) without obesity, 3.5 (95% CI, 1.2 to 12.7) with cardiovascular disease, 3.7 (95% CI, 1.4 to 11.7) without respiratory disease, 3.5 (95% CI, 1.4 to 10.7) without diabetes, and 4.3 (95% CI, 1.7 to 13.9) without hyperlipidemia. The relationship between logPAI-1 levels and mortality was evident in patients, irrespective of obesity status, and in those with cardiovascular disease, and those without, respiratory disease, diabetes, and hyperlipidemia.

## Discussion

These investigations demonstrate that PAI-1 is an independent poor prognostic factor for COVID-19. The results are consistent with those reported by Zuo et al.^14^.

Studies using experimental models of lung injury report that elevated PAI-1 levels can initiate lung injury^15,16^. Therefore, drugs targeting PAI-1 are expected to be effective to treat COVID-19 pneumonia. Hirai et al. reported the efficacy of a plasminogen activator inhibitor-1 inhibitor (TM5614) in patients with mild to moderate COVID-19^6^.

These results are expected to facilitate early detection and therapeutic intervention in patients prone to severe disease and facilitate the selection of patients for whom PAI-1 inhibitors could be used for treatment. Furthermore, it could potentially contribute to the efficient allocation of finite medical resources and positively impact healthcare economics.

This study has some limitations. First, it is single-center and the sample size small. Furthermore, given that this is a chart review study, pertinent clinical information, such as other comorbidities (chronic liver disease, autoimmune or rheumatologic disorders, and hematologic conditions), may have been incomplete, thereby affecting the accuracy of the prevalence of the comorbidities catalogued.

In summary, PAI-1 levels are suggested to be an independent factor influencing COVID-19 severity. By measuring PAI-1 levels at the initial visit, it may be possible to screen patients who are likely to develop severe disease. The promoter site single nucleotide polymorphism (SNP) 4G/5G is known to correlate with plasma levels of PAI-1. Our previous studies show that the 4G/5G polymorphism can be a genetic marker of asthma severity, and PAI-1 levels can be used as a novel treatment option for uncontrolled asthma^17^^18^. Others have examined the association between 4G/5G polymorphism and COVID-19 severity^19^. These studies and ours suggest that PAI-1 SNP can be used as a biomarker for severe COVID-19 together with PAI-1 plasma levels. Further clinical studies are needed to determine if these biomarkers may identify patients who may develop severe disease and potentially benefit from PAI-1-targeted therapies.

## Data Availability

The datasets used and/or analyzed during the current study available from the corresponding author on reasonable request.
